# Present and Future Therapy of *Cryptococcus* Infections

**DOI:** 10.3390/jof4030079

**Published:** 2018-07-03

**Authors:** Ahmad Mourad, John R. Perfect

**Affiliations:** Division of Infectious Diseases, Department of Medicine, Duke University Medical Center, Durham, NC 27710, USA; ahmad.mourad@duke.edu

**Keywords:** *Cryptococcus*, cryptococcosis, cryptococcal meningitis, antifungal therapy, antifungal agents

## Abstract

Cryptococcal infections burden the immunocompromised population with unacceptably high morbidity and mortality. This population includes HIV-infected individuals and those undergoing organ transplants, as well as seemingly immunocompetent patients (non-HIV, non-transplant). These groups are difficult to manage with the current therapeutic options and strategies, particularly in resource-limited settings. New trials aimed at providing the best treatment strategies for resource-limited countries that will reduce costs and adverse reactions have focused on decreasing the length of therapy and using more readily accessible antifungal agents such as fluconazole. Furthermore, the emergence of antifungal resistance poses another challenge for successful treatment and may require the development of new agents for improved management. This review will discuss the principles of management, current and future antifungal agents, as well as emerging techniques and future directions of care for this deadly infection.

## 1. Introduction

*Cryptococcus* spp. are encapsulated, polysaccharide-coated yeasts with two major species (*C. neoformans* and *C. gattii*) known to cause disease in humans. These *Cryptococcus* spp. can invade the central nervous system (CNS) causing fungal meningoencephalitis. The global incidence of cryptococcal meningitis was recently estimated at approximately 220,000 per year [1]. Certain immunocompromised hosts are at particularly high risk of cryptococcal meningitis. For instance, 15% of AIDS-related deaths in 2014 were due to cryptococcal meningitis [1]. With present therapies, mortality associated with cryptococcal meningitis is unacceptably high in both developed and developing countries. One-year mortality for those receiving care was estimated at 70% in low-income countries, 40% in middle-income countries and 20–30% in Europe and 20% in North America for 2014 [1]. The introduction of highly active antiretroviral therapy (HAART) for HIV has reduced rates of fungal disease. However, the increasing number of transplant recipients and patients receiving immunosuppressive medications such as corticosteroids, new biological drugs and new anti-neoplastic therapies has created an at-risk population with a high incidence of cryptococcal infection [2].

At present, antifungal drugs such as polyenes, flucytosine (5-FC), triazoles, and their combinations are the gold standard of care for cryptococcal meningitis [3]. However, the development of immunotherapeutic drugs, neurapheresis, novel targeted compounds and the repurposing of existing medications might change the way we will manage cryptococcal meningitis in the future. This review will discuss the important drugs, their strategies for application, and future directions of care for this deadly infection.

## 2. Current Therapeutic Options

### 2.1. Antifungal Agents

A three-part regimen of “induction-consolidation-maintenance” is the favored therapeutic strategy for cryptococcal meningitis. Induction therapy is currently recommended with intravenous (IV) amphotericin B (deoxycholate): (0.7–1 mg/kg/day), or liposomal amphotericin B: (3–4 mg/kg/day), or amphotericin lipid complex: (5 mg/kg/day) and oral 5-FC (100 mg/kg/day) for at least 2 weeks [3]. IV formulations of 5-FC can be administered in some severe cases or in those unable to tolerate oral intake, if available. The length of the induction period (2–4 weeks) can be extended in patients with poor initial response, those with worse prognoses or those with non-HIV, non-transplant underlying disease. The recent “Advancing Cryptococcal Meningitis Treatment for Africa” (ACTA) trial showed that one week of amphotericin B deoxycholate plus 5FC had the lowest mortality among the treatment arms (including a control arm of the standard two weeks of combination therapy). The “AmBisome plus high dose fluconazole for treatment of HIV-associated cryptococcal meningitis” (AMBITION-cm) trial has shown that shorter durations of high dose liposomal amphotericin B and fluconazole are non-inferior to the standard two-week induction course, including a single high dose (10 mg/kg/day) of liposomal amphotericin B [4,5]. It is important to emphasize that these studies relate to resource-limited areas and their need to find cost-effective therapies. In contrast, the use of lipid formulations of amphotericin B in induction therapy is favored in resource-available areas for all patients because of their better safety profile. This is especially important in patients already at a high risk for nephrotoxicity occurrence with polyene treatment such as transplant recipients and those receiving concomitant nephrotoxic drugs [6]. Some studies have investigated the pharmacokinetics of liposomal amphotericin B, to reduce the induction period while maintaining efficacy. The pharmacodynamic effects, measured by reduced fungal burden in non-immunocompromised murine models of cryptococcal meningitis, of a single high dose of liposomal amphotericin B (20 mg/kg) were the same as a longer dosing period of 20 mg/kg/day for 2 weeks [7]. This suggests that shorter courses of liposomal amphotericin B may be effective. However, it is important to consider that these short courses of induction therapy performed in resource-limited areas may not be relevant to resource-available sites where more precise monitoring of patients allows for a longer effective induction period.

In resource-limited areas, fluconazole can be substituted for 5FC at doses of ≥800 mg/day due to its availability and this also captures the potential advantages of combination therapy during the induction therapy period [8]. Even the use of the oral combination regimen of 5FC and fluconazole is apparently not inferior to the polyene-containing regimens if intravenous drugs in resource-limited settings are difficult to administer [4]. The use of fluconazole as induction monotherapy at higher doses (1200 mg/day) is not favored as it is associated with increased mortality, but high dose fluconazole (1200 mg/day) with 5FC (100 mg/kg/day) may be utilized [4,9]. It is particularly frustrating that most of the population affected by cryptococcosis does not have access to 5FC and those with access may pay exorbitant prices [10,11].

High dose fluconazole (800 mg/day) has also been shown to be an effective azole for the 8-week consolidation phase following induction therapy [8]. Fluconazole (200 mg/day) is also used for the final phase of therapy, maintenance, for 6–12 months in non-HIV infected patients and at least 12 months in HIV patients. In patients with HIV, maintenance with fluconazole is discontinued after HAART has been reintroduced following a CD4 count ≥100 cells/μL and undetectable HIV RNA ≥3 months after at least a year of therapy [3].

Fluconazole has also been used effectively in a strategy that combines cryptococcal antigen screening and pre-emptive therapy. HIV-infected patients with a CD4 count ≤100 cells/μL are screened for presence of serum cryptococcal antigen (CrAg) using lateral flow assays and, if positive, pre-emptive antifungal therapy with oral fluconazole will be started [12]. If CNS symptoms are present or serum CrAg titers are ≥1:160, a lumbar puncture should be performed to assess for evidence of cryptococcal meningitis [12]. Survival with this strategy was dramatically improved, with a 30-month survival of 71% for those who received fluconazole and later developed cryptococcal meningitis while the 2-month survival was 0% in those CrAg positive patients who developed cryptococcal meningitis and had not received fluconazole [13]. With the worldwide incidence of cryptococcal antigenemia estimated at over a quarter million in HIV-infected patients with low CD4 counts, this pre-emptive strategy has proven to be cost-effective in decreasing the incidence of cryptococcal disease and improving survival in countries with high prevalence of the disease [13]. However, recent findings have indicated that this pre-emptive strategy with fluconazole may not be adequate in those with higher serum CrAg titers (≥1:160) and there is a need to determine the best treatment strategy for these patients who will likely require a diagnostic lumbar puncture and possibly induction therapy [14].

For mild to moderate pulmonary cryptococcosis, as well as other extra-CNS and non-disseminated cryptococcal infections, fluconazole monotherapy is currently recommended [3]. Other triazoles such as itraconazole, posaconazole and isavuconazole have been used successfully to treat cryptococcal meningitis in some instances. Due to either their poor CNS penetration, issues with bioavailability or lack of experience, these antifungals have generally been considered less reliable [15,16,17]. Voriconazole has also had some success, mostly in non-immunocompromised hosts [18].

### 2.2. Corticosteroids 

Systemic corticosteroid use is a potentially life-saving intervention to be considered for the management of Immune Reconstitution Inflammatory Syndrome (IRIS) in cryptococcal meningitis, with a dose taper over 2–6 weeks [3]. However, precision of dosing, duration of therapy, or whether prednisone or dexamethasone is used, are uncertainties that require further study. The use of corticosteroids for routine management of cryptococcal management is not recommended. A trial was terminated due to increased mortality and adverse events, as well as reduced fungicidal activity of the antifungal agents, in a group of HIV-infected patients receiving adjuvant dexamethasone [19].

### 2.3. Lumbar Puncture

In some cases of cryptococcal meningitis, the clumping of fungal cells in and around the arachnoid villi may cause obstruction of the dynamic outflow of cerebrospinal fluid (CSF), resulting in increased intracranial pressure (ICP). Increased ICP is associated with higher mortality in patients with cryptococcal meningitis [20]. Lumbar puncture (LP) is currently recommended to relieve ICP [3]. Some studies have suggested the potential benefit of a therapeutic LP in all patients with cryptococcal meningitis. An improvement in survival by nearly 70% was associated with therapeutic LP, independent of increased ICP in resource-limited areas [21]. Some studies and case reports have shown that using a schedule of serial LPs in patients with cryptococcal meningitis significantly reduced mortality as well as complications of elevated ICP, such as hearing loss and blindness [22,23]. Although the optimal frequency and specific indications for repeating LP have yet to be defined, it should be considered in the management of all cryptococcal meningitis patients with persistent symptoms of increased ICP.

## 3. Future Therapeutic Options and Directions

With innate cryptococcal resistance to antifungal compounds such as echinocandins and the recent reports of acquired clinical resistance of cryptococcal strains to antifungal agents such as fluconazole, some treatment dilemmas emerge [24,25]. These may include the use of higher doses of antifungal agents, causing a potential increase in drug-related adverse events. The need for new antifungal agents (Table 1) as well as improved strategies and techniques are critical for better management of cryptococcal disease.

### 3.1. New Antifungal Agents

*APX001* represents a new class of antifungal agents that inhibit the fungal protein Gwt1, destabilizing the fungal cell wall. Compared to either agent alone, a combination of APX001 and fluconazole significantly reduced fungal burden in a non-immunocompromised murine model of cryptococcal meningitis [26]. A recent series of APX001 congeners have been synthesized and some of these compounds have shown very impressive direct fungicidal activity against *Cryptococcus* spp. in vitro and yeasts within the murine CNS [27]. Oral APX001 is currently being tested in a Phase I clinical trial (NCT03333005) in neutropenic patients with Acute Myeloid Leukemia (AML) and invasive candidiasis. This class of antifungal compound may perform extremely well in treating cryptococcosis that responds well to efficient fungicidal activity. These new congeners may provide a major advance in anticryptococcal therapy.

AR12 inhibits fungal acetyl-CoA synthetase and has antifungal activity against *Cryptococcus* spp. both in vitro and in vivo. AR12 also improves the activity of fluconazole in non-immunocompromised murine models of cryptococcosis [28]. This celecoxib-derivative anticancer agent has been previously tested previously in a phase I clinical trial.

T2307 is an allylamine that displays significant anticryptococcal activity in vitro and in vivo by collapsing mitochondrial membrane potential. It has very potent anticryptococcal activity against *C. gattii* in non-immunocompromised murine models of cryptococcosis [29].

V-T1129 and VT-1598 are two new azoles (tetrazoles) in development that have shown promising anticryptococcal activity in non-immunocompromised murine models and have reduced interactions with host CYP450 cytochromes [30,31]. They showed higher potency than all other clinically approved azoles in a murine model. VT-1129 also has anticryptococcal activity against fluconazole-resistant clinical isolates of *C. neoformans*, providing a possible alternative in such azole-resistant cases [30].

Acylhydrazones such as BHBM target the non-mammalian sphingolipid pathway, which is critical for the virulence of *C. neoformans* and *C. gattii*. Three of nineteen derivatives of BHBM display potent in vitro activity against *C. neoformans* and one derivative showed antifungal activity with suitable CNS penetration in a non-immunocompromised murine model [32].

Ilicicolin H is a polyketide that inhibits fungal mitochondrial cytochrome bc1 reductase with high selectivity. It has potent in vivo activity in a non-immunocompromised murine model of disseminated cryptococcosis [33].

Derivatives of the naturally-occurring compound *Sampangine* have in vitro antifungal activity against *C. neoformans*. However, the mechanism of action of these compounds is not known and their pathway to development is uncertain [34].

### 3.2. Repurposing Drugs to Treat Cryptococcosis

Sertraline, a Selective Serotonin Reuptake Inhibitor (SSRI), has potent fungicidal activity against *C. neoformans* in vitro. It inhibits protein synthesis by interacting with fungal translation factors [35]. It acts synergistically as an adjunct to fluconazole therapy in non-immunocompromised murine models of systemic cryptococcosis and initial studies suggested that it may help increase CSF clearance of *Cryptococcus* in human subjects [36]. A recent clinical trial (NCT01802385) assessing its use with amphotericin B-based regimens for cryptococcal meningitis showed improved rates of clearance of CSF Cryptococci [37].

The calcineurin pathway is critical in cryptococcal virulence. Use of calcineurin inhibitors, such as tacrolimus or cyclosporine to target fungal calcineurin synergistically improved antifungal activity of both amphotericin B and fluconazole [38]. Since many solid organ transplant recipients receive calcineurin inhibitors to produce immunosuppression and prevent organ rejection still develop cryptococcosis, there is a need to find a concomitant congener where immunosuppression is dialed down but antifungal adjuvant activity persists. If so, this target and its blocking-molecules may help prevent and/or treat cryptococcosis.

The estrogen receptor antagonists, Tamoxifen and Toremifene have shown synergistic activity with fluconazole and amphotericin B against *C. neoformans* in vitro, and together with fluconazole in a non-immunocompromised murine model of cryptococcosis [39]. They function by inhibiting the formation of the calmodulin-calcineurin complex [39]. A phase II clinical trial assessing the use of tamoxifen in patients with cryptococcal meningitis (NCT03112031) is in progress.

Heat Shock Protein 90 (HSP90) is an important regulator of cryptococcal pathogenicity and has been shown to localize to the fungal cell wall [40]. Thus, inhibitors of HSP90 could serve as compounds for future therapeutic development. The HSP pathway may be used to attack *Cryptococcus* spp., with a series of these HSP90 inhibitors already being studied [40].

Finally, the protease inhibitors, such as those used for antiretroviral therapy, have been suggested to impact the production of proteases that might affect cryptococcal virulence. However, for *C. neoformans*, the clinical significance of these inhibitors or the importance of the proteases they block have yet to be determined [41].

### 3.3. Immunotherapy, Monoclonal Antibodies and Vaccines

The increased release of intrinsic host immune modulators in response to cryptococcal infection has been consistently shown to be associated with survival of the host. For example, the HIV-infected individuals who were treated for cryptococcal meningitis and survived, had higher levels of several immune modulators in the CSF (IFNγ, TNF-α, IL-6 and IL-8) compared to those who did not [42]. These observations set the stage for focus on IFNγ. IFNγ modulates host immunity by stimulating the Th1 response against *Cryptococcus* spp. Th1 is important in the control of cryptococcal infection in many systems [43]. Therefore, the role of IFNγ as an adjunct to antifungal therapy has been investigated in trials of HIV-infected patients with cryptococcal meningitis [44]. Fungal clearance from the CSF was increased when IFNγ was combined with standard amphotericin B therapy, but these studies were insufficiently powered to prove a survival advantage [43]. Routine use of recombinant IFNγ therapy has not been recommended in guidelines but remains an alternative recommendation for patients with refractory cryptococcal infection [3]. Hesitation about its use likely resides in the lack of precision in the control of immunity in a disease where Immune Reconstitution Inflammatory Syndrome (IRIS) can be deadly. Research is needed to determine the value and utility of IFNγ prospectively and precisely in routine therapy.

Monoclonal antibodies are more frequently being utilized for targeted therapies, with the majority used as anticancer drugs. Targeted therapy has the advantage of limiting cross reactivity with normal human host components. Monoclonal antibody therapy in treating cryptococcal infections has a rich history built on the identification of both protective and non-protective antibodies for cryptococcosis [45]. The most potent, anticapsular cryptococcal antibodies were protective in concert with antifungal agents in mice, and the monoclonal anticryptococcal antibody (MAb 18B7) reduced serum cryptococcal antigen titers in HIV-infected patients treated successfully for cryptococcal meningitis [46,47].

β-glucan is a unique and important component of the fungal cell wall and is essential for cryptococcal survival. Targeting β-glucan with monoclonal antibodies such as MAb 2G8 inhibits cryptococcal growth in vitro and significantly reduces fungal burden in an immunocompromised murine model of cryptococcosis [48]. Melanin is another essential cryptococcal cell wall component. It assists virulence by shielding fungal cells from host immune responses such as macrophage oxidative damage. Cryptococcal infection stimulates host production of antibodies against melanin [49]. Monoclonal antibodies against melanin slow growth of *C. neoformans* in vitro and prolong survival in infected non-immunocompromised murine models of cryptococcosis [50].

Monoclonal antibodies have been used to deliver radioimmunotherapy to yeast cells. This uses monoclonal antibodies tagged with radiation-emitting particles to deliver toxic levels of radiation to specific cells and induce cell death, with limited effect on bystander cells. This strategy has been approved for use in targeting certain tumor cells and explored to target *Cryptococcus* spp. Radiolabeled antibodies directed against *C. neoformans* polysaccharide capsule almost completely eliminated *C. neoformans* in non-immunocompromised murine models of cryptococcosis [51]. In comparison, treatment with systemic amphotericin B does not significantly reduce fungal burden. A subsequent study showed that the radiolabeled antibodies did not affect mammalian cell survival [52].

Vaccination with conjugate vaccines directed against cryptococcal capsule polysaccharides improved survival in non-immunocompromised mice chronically infected with systemic *C. neoformans* [53]. Furthermore, naïve mice inoculated with the sera of vaccinated mice had prolonged survival when subsequently infected with *C. neoformans* [54]. There are other potent cryptococcal antigens, such as chitin deacetylases, which can be used to deliver vaccine protection [55]. Even the use of a live, IFNγ-producing *C. neoformans* strain has been shown to stimulate protective host immunity [56]. Vaccination of high-risk patient populations may ultimately prove to be a preventative strategy for cryptococcal disease control, but it will be critical to select the most appropriate population to make the vaccine cost-effective.

### 3.4. Novel Techniques

Neurapheresis therapy is a new technique for the management of cryptococcal meningitis under investigation. A porous membrane is used to filter yeasts and a pump circulates and reintroduces filtered CSF back into the subarachnoid space. The extracorporal filtration of CSF rapidly removes yeast and cryptococcal antigen from the subarachnoid space. In an immunocompromised rabbit model of cryptococcal meningitis, neurapheresis cleared fungal cells ≥5 μm in diameter from the CSF [57,58]. Several cycles of filtration gave a substantial decrease in the numbers of viable cryptococcal yeast cells from the CSF, as well as a reducing CSF antigen levels [57,58]. Neurapheresis potentially provides a three-fold advantage in the management of cryptococcal meningitis: 1. a rapid decrease of fungal burden in the subarachnoid space; 2. a reduction in or prevention of elevated ICP; 3. delivery and circulation of antifungal agents such as amphotericin B directly into the subarachnoid space.

## 4. Conclusions

Cryptococcal disease remains difficult to manage, with the high morbidity and mortality resulting from its invasion into the CNS. The complications arising from this infection are important to recognize and treat. Early diagnosis and therapy with an appropriate fungicidal regimen is needed to give the best outcome. However, treatment strategies must adapt to the restrictions faced in resource-limited areas and the emergence of drug resistance. The development of new strategies utilizing more readily available and less toxic drugs are needed for the effective management of cryptococcosis.

## Figures and Tables

**Table 1:** New anticryptococcal agents in development.

New Agents	Mechanism of Action	Potential Utilization Strategy
APX001	Inhibits the fungal protein Gwt1	Combination with existing antifungal agents, such as fluconazole
AR12	Inhibits fungal acetyl-CoA synthetase	Combination with existing antifungal agents such as fluconazole
T2307 (allylamine)	Collapses the mitochondrial membrane potential	
VT-1129, VT-1598 (tetrazoles)	Inhibits ergosterol biosynthesis	Treatment of fluconazole-resistant *Cryptococcus* strains
BHBM (Acylhydrazones)	Targets non-mammalian sphingolipid pathway	
Ilicicolin H (polyketide)	Inhibits fungal mitochondrial cytochrome bc1 reductase	
Sampangine derivatives	Unknown	
